# Adverse Effects of Pregnancy on Keloids and Hypertrophic Scars

**DOI:** 10.7759/cureus.12154

**Published:** 2020-12-18

**Authors:** Nada E Ibrahim, Shazrinizam Shaharan, Baljit Dheansa

**Affiliations:** 1 Plastic and Reconstructive Surgery, Leicester Royal Infirmary, Leicester, GBR; 2 Plastic Surgery, Queen Victoria Hospital NHS Foundation Trust, East Grinstead, GBR

**Keywords:** keloid scar, hypertrophic scar, pregnancy effects on scars, ­scars and keloids, puberty effects on scars

## Abstract

There are many well-known risk factors for keloids and hypertrophic scars (HTS) including ethnicity, family history, and history of previous keloids or HTS. An association, which has been previously observed, exists between pregnancy and growth and worsening of keloid and HTS. This association is less well known amongst physicians and less documented in the literature.

In this paper we discuss two cases of extreme worsening of keloid scars during pregnancy. We have also witnessed the transformation of a pre-existing scar into a keloid scar during puberty. We attribute this to the hormonal effects of pregnancy and puberty hormones which could potentially trigger the growth of pre-existing keloids and HTS.

This may have an impact on many patients and we therefore recommend women and girls who have keloid and hypertrophic scarring be made aware of this potential effect.

## Introduction

Despite the fact that it is nearly 1000 years since the first documented historical reference to keloid scar formation [1], it still remains one of the most frustrating and poorly understood dilemmas facing health care professionals dealing with skin conditions. Many theories have been propounded in trials to reveal the underlying pathological process behind keloids and hypertrophic scar formation. It is believed that external injury acts as a triggering factor for a local inflammatory process resulting in a fibro-proliferative disorder and abnormal healing characterised by high collagen synthesis and deposition [2].

Risk factors for keloids and hypertrophic scarring include black, Hispanic, or Asian descent. Previous history and family history of keloids also increase the chances of developing keloids and HTS. Pregnancy and hormonal effects during puberty have been alluded to as risk factors for worsening of keloid scars [3-4]. This latter association is less well known amongst physicians and may have an impact on many patients.

## Case presentation

In our practice, we have witnessed two extreme cases of worsening and recurrence of keloid scars during pregnancy. The first case is a lady of Caucasian origin who has been under our care over the last 12 years. She had an injection to her left shoulder at the age of nine. A few years later as she was going through puberty the scar went through a growth spurt and transformed into a thick keloid scar. This was managed initially, in another centre before the patient was referred to us, by excision, split skin grafting and radiotherapy.

**Figure 1:**
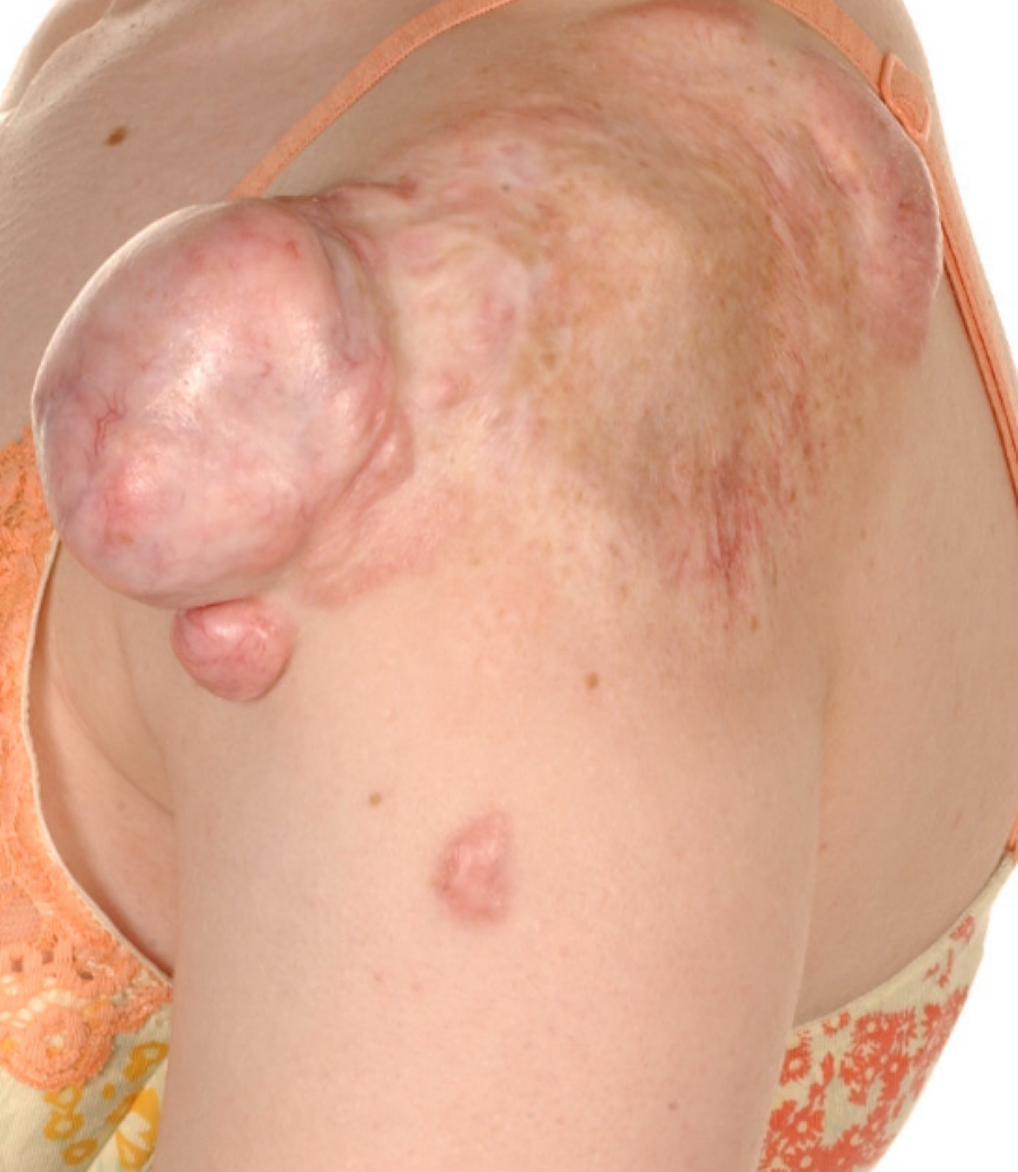
Keloid Scar Left Shoulder Patient 1 showing keloid scar left shoulder which has flourished to size of a tennis ball during pregnancy.

The scar initially responded to treatment and remained settled. Then many years later at the age of 21, the patient became pregnant and the keloid scar went through another growth spurt, but a rather significant one, and reached a considerable size, almost a size of a tennis ball (Figure 1). Apart from the pregnancy, we could not identify any other risk factor to explain this significant growth spurt. We could not ignore the question of whether we can attribute this to the hormonal changes that could have possibly stimulated the scar growth.

**Figure 2:**
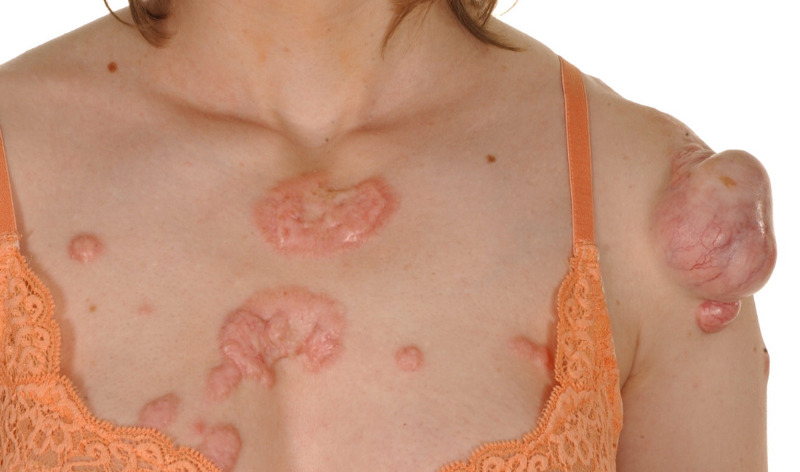
Keloid Scars on Anterior Chest Wall Patient 1, picture showing keloid scars on anterior chest wall and left shoulder which have progressed during pregnancy.

The patient had also developed some keloid scarring on her anterior chest wall as a result of minor trauma. These scars also grew during the pregnancy, however to a lesser extent compared to her left shoulder scar. These scars were quite flat before the pregnancy and then continued to grow throughout the pregnancy and became raised as shown in Figure 2 below.

**Figure 3:**
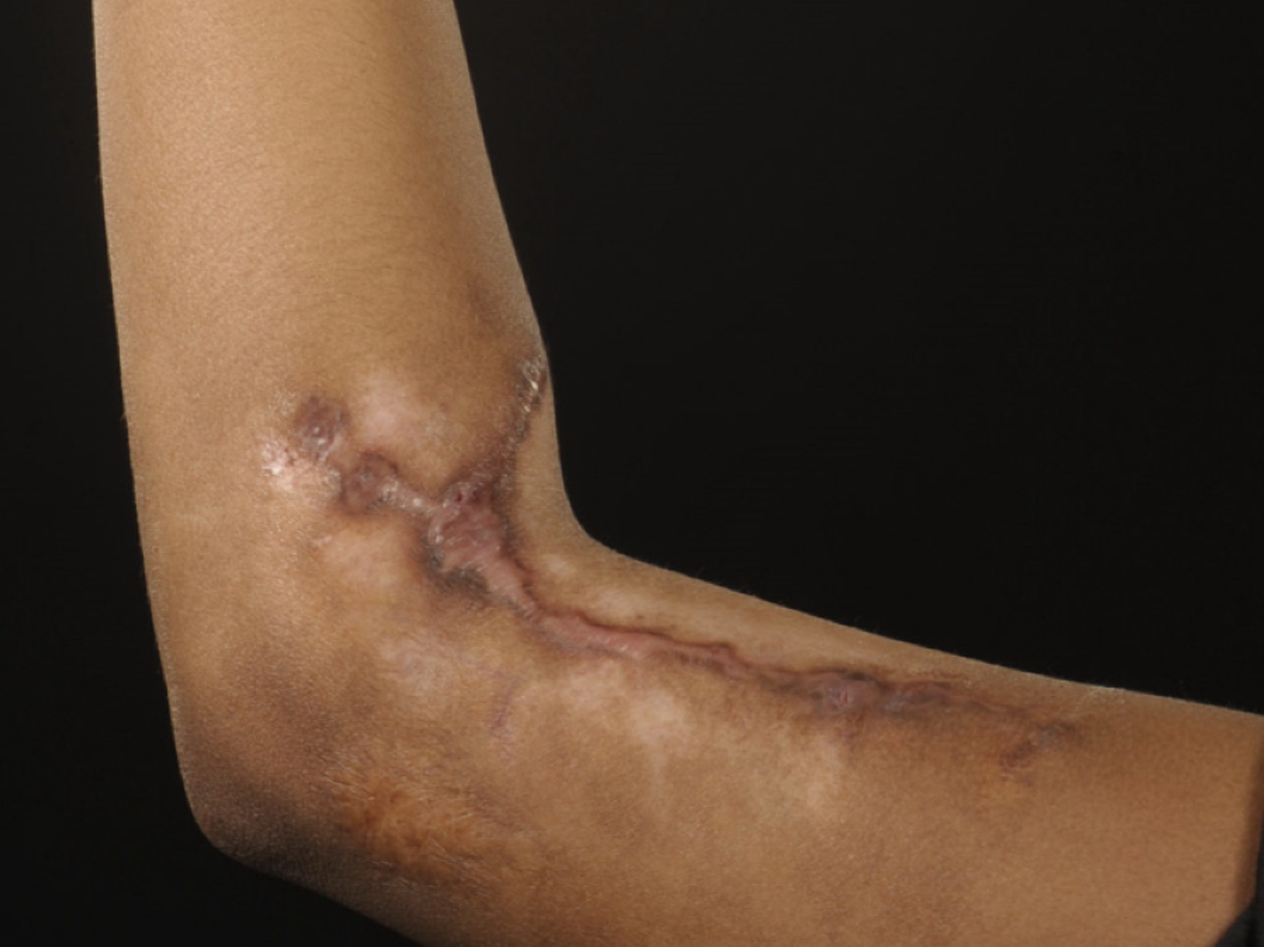
Scar Left Elbow Patient 2's left elbow scar two years after excision showing flat scar with no recurrence.

The second case was a lady of Afro-Caribbean descent who sustained a burn to her left elbow antecubital fossa as a child. This later developed into a keloid scar. The scar was managed by excision and multiple steroid injections. The scar remained under control with treatment and ongoing pressure garment (Figure 3).

**Figure 4:**
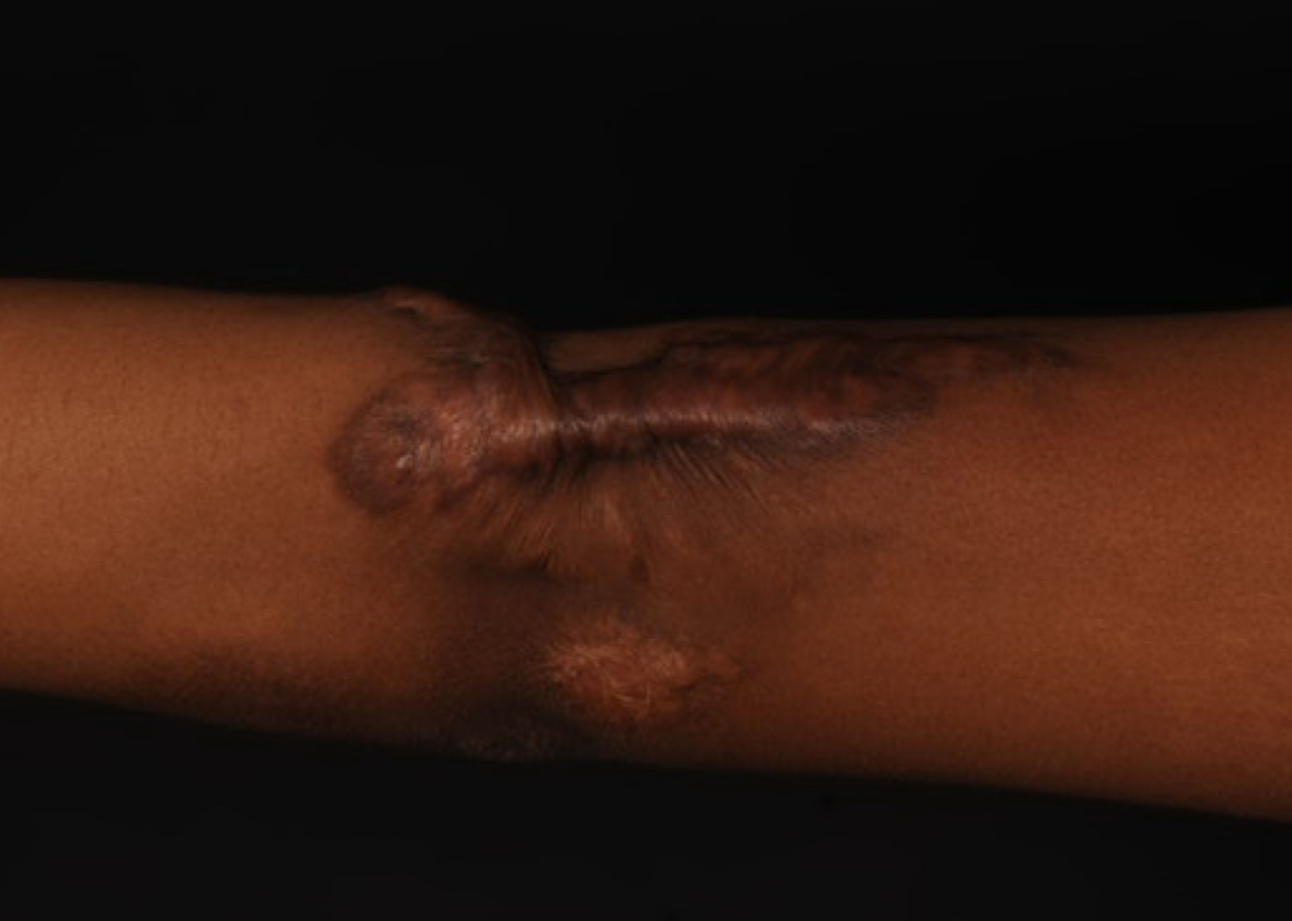
Keloid Scar Recurrence During Pregnancy Patient 2; scar left elbow showing recurrence and activity of keloid scar during pregnancy. Photo taken while patient was in 1st trimester of pregnancy.

Years later she became pregnant and started to notice significant worsening the scar. Early in her first trimester the scar started to become painful, itchy, thicker and wider. Again, a significant growth spurt in the keloid was observed and we attributed it to the effect of pregnancy hormones on the keloid scar (Figure 4).

Management

For the two patients above, active keloid scar management in the form surgical excision and steroid injections were deferred until after delivery. The efficacy of any intervention during the pregnancy was questionable with the presumed ongoing effects. Furthermore, there was a big safety question about repeated steroid injections during pregnancy for such large lesions that would require high doses of intralesional steroid injections. All conservative non-interventional measures like pressure garments and scar massaging were continued during pregnancy. The keloid scars for both patients continued to become symptomatic throughout pregnancy with continuous growth in size. 

**Figure 5:**
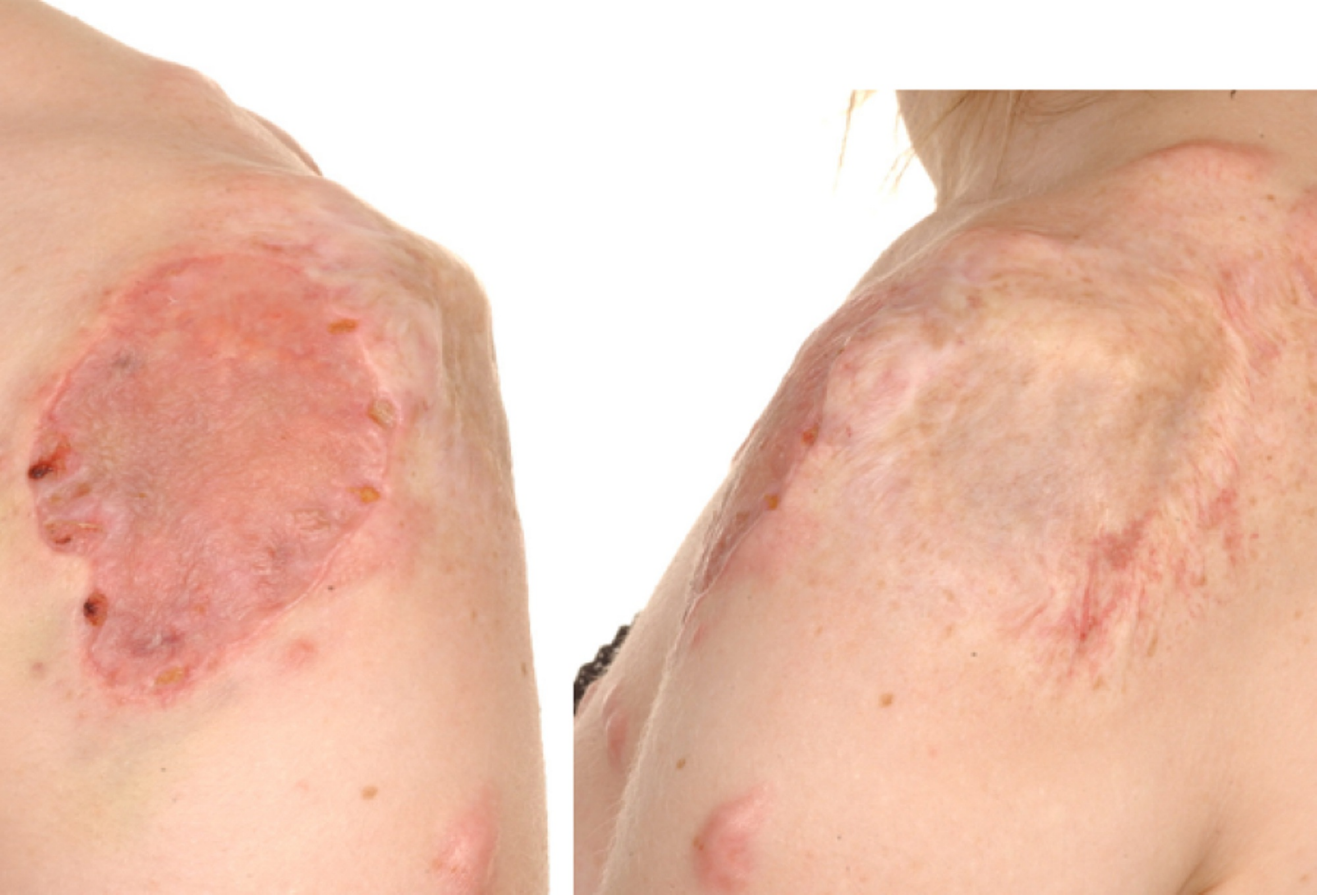
Skin Graft Following Excision of Keloid Scar on Left Shoulder Patient 1; keloid scar on left shoulder treated with excision and split skin graft. Picture showing graft healing and maturing well.

**Figure 6:**
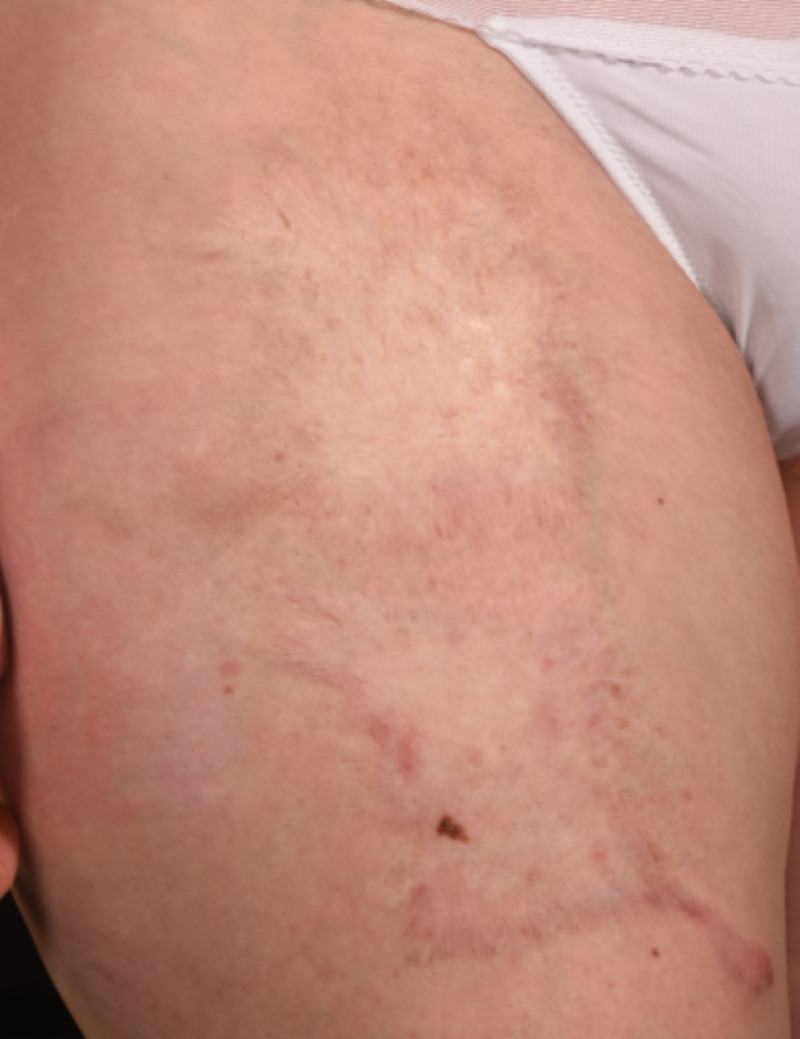
Skin Graft Donor Site Picture showing skin graft donor site for Patient 1 ten years following excision and grafting of her keloid scars.

**Figure 7:**
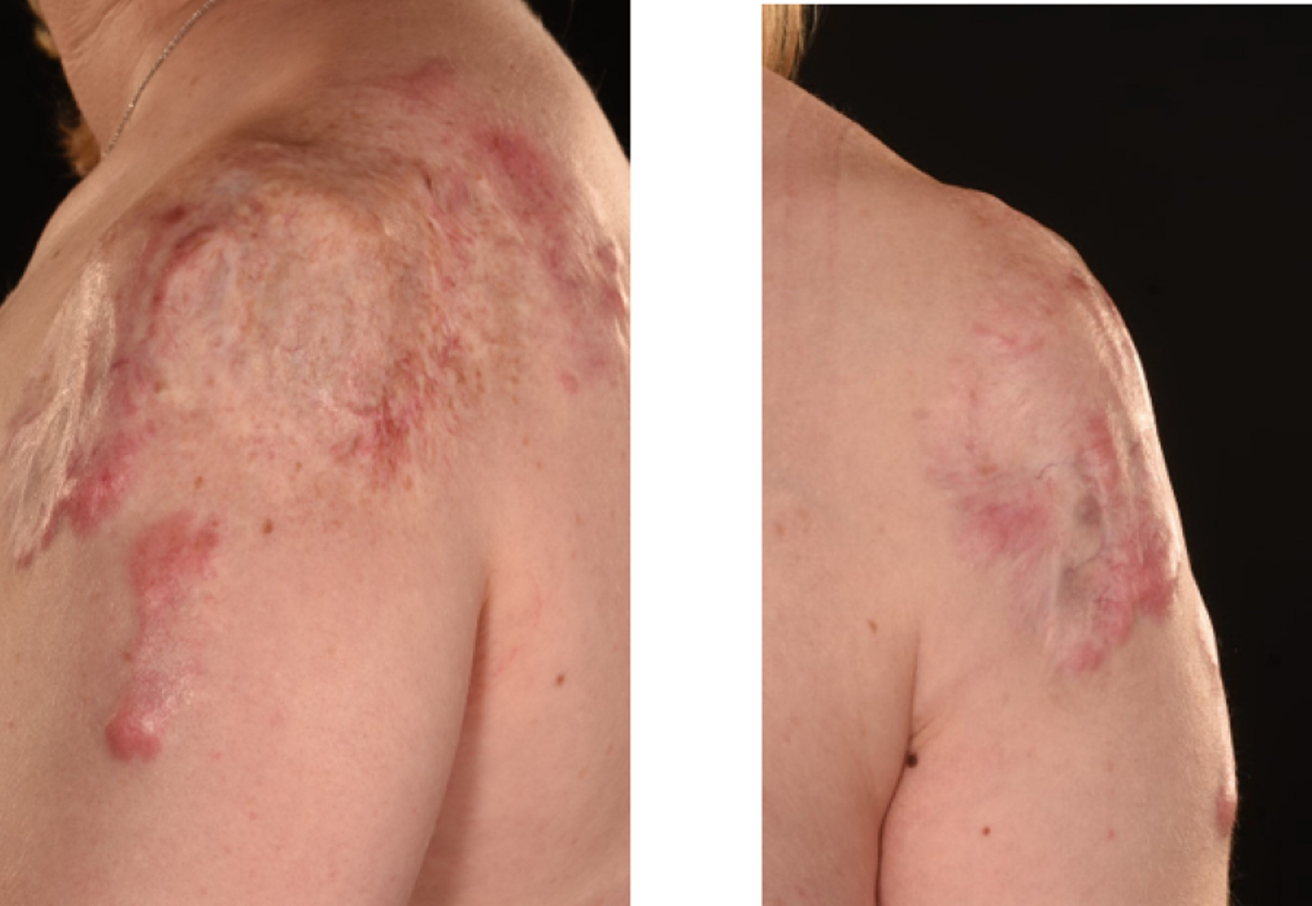
Skin Graft and Scars 10 Years Later Picture showing Patient 1 scars remaining subtle 10 years following excision and grafting of keloid scars on her left shoulder.

After delivery, patient 1's extensive keloids were managed with multiple excisions, split skin grafting and multiple ongoing steroid injections. The keloid scarring on her left shoulder has healed and settled well following the excision and grafting (Figure 5). The donor site has also healed well (Figure 6) with very minor hypertrophic scarring at one edge. This has significantly settled with steroid injections. She has also been receiving multiple steroid injections to manage the keloids on her anterior chest wall. Over the years the graft remained settled on her right shoulder with no recurrence (Figure 7).

**Figure 8:**
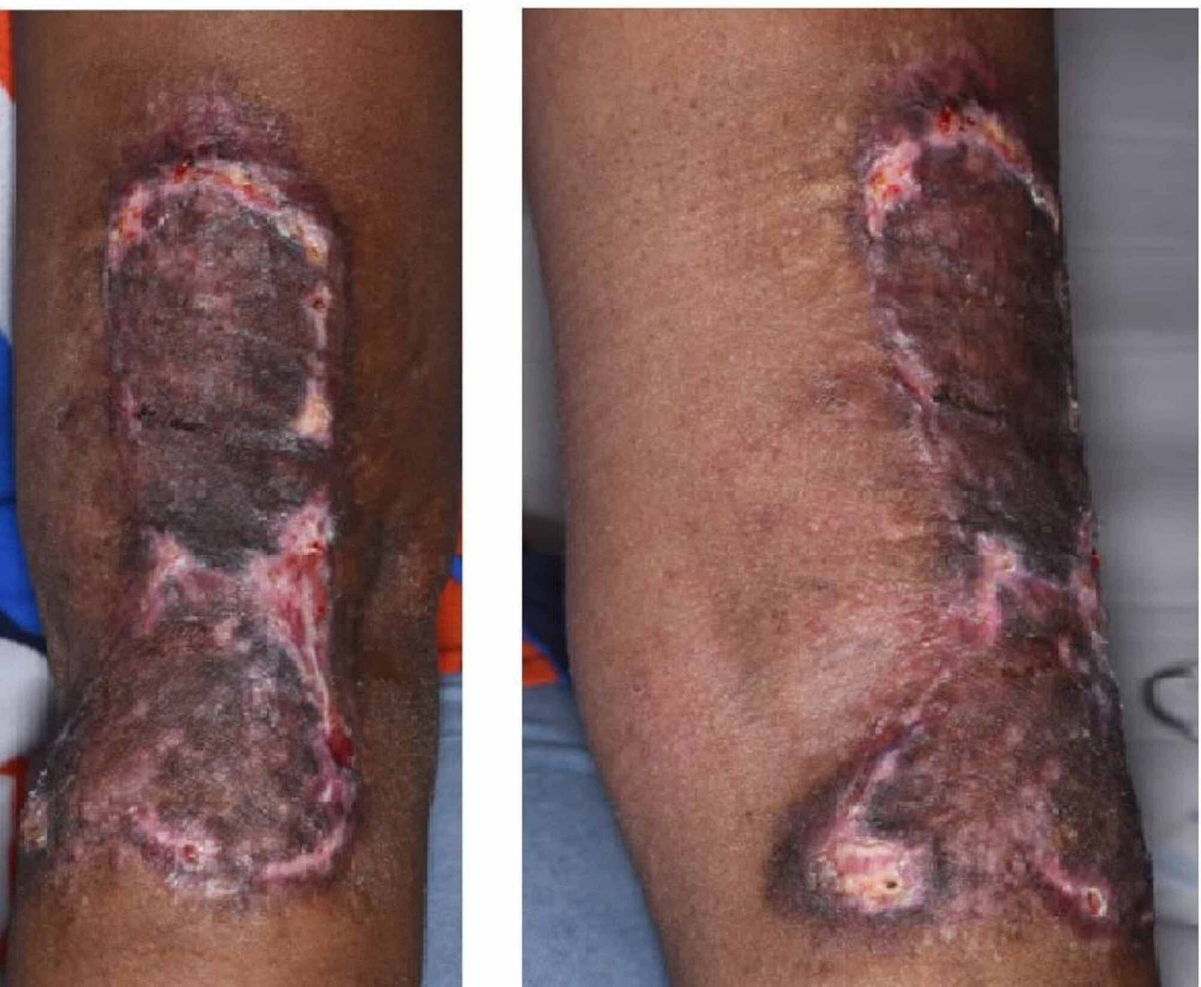
Graft on Left Elbow Picture showing skin graft six weeks post operatively on Patient 2 following excision of her keloid scar on left elbow.

Patient 2 was also managed by excision and grafting. She had a full thickness skin graft harvested from her groin area. Her graft has taken well (Figure 8). It is still early in her case to ascertain her long-term outcome.

## Discussion

In these two cases a very apparent link was noticeable between the effect of puberty and pregnancy leading to the growth and worsening of the keloid scars. The link can not be ignored specially in the lack of any other identifiable risk factors for the growth of the very well settled scars. We would like to attribute this to the hormonal changes that could have possibly stimulated the growth of the scars. However there is very little evidence in the literature to support this theory.

In the literature there are few reported cases of worsening and recurrence of keloids during pregnancy [3-4], however no powerful studies of level 1 evidence to ascertain the association. In a study of connective tissue tumours, Geschichter and Lewis bio-assayed a single keloid of an ear that had been preserved in formalin; they reported that this keloid tissue contained large amounts of oestrogen and gonadotropic substances [5]. However, no confirmation of their work has appeared in the literature.

Vargas, while injecting oestrogen in castrated female rhesus monkeys in an attempt to induce formation of fibroids, reported a keloid plaque over the stomach in one animal and another had pronounced operative adhesions and fibrosis at the laparotomy site [6]. Although this might not be statistically significant the correlation cannot be ignored, and further studies are required to fill in the knowledge gaps.

Not only do pregnancy and puberty appear to be associated with worsening of a pre-existing keloid or hypertrophic scar, but we have also noted, in patient 1, that puberty has triggered a previous scar to grow into a keloid scar. Jacobsson observed a similar effect when reporting a case of a woman whose four-year-old scar became hypertrophic during pregnancy [7]. Cosman et al. reported similar changes in another case [8].

Although keloids and hypertrophic scars may develop at any age, they are more often witnessed between the age of 10 and 30 years and they tend to resolve during menopause [9]. This interesting observation makes us suspicious that it has the same basis as the reactivation and worsening observed during puberty and pregnancy.

Management of keloids and hypertrophic scars during pregnancy remains a virgin area with very little evidence and guidance. Many areas need to be addressed regarding the safety of various treatment methods (steroids and surgical excision) during pregnancy. The effectiveness of treatment during pregnancy with the ongoing impact of hormones is also questionable. In almost all of the cases reported a similar approach of delaying surgical treatment and steroid injections until after delivery.

## Conclusions

We conclude that there is a noticeable increase in the activity, in the form of worsening or recurrence, of previously settled keloid scars during pregnancy and puberty in some patients. We attribute this to the hormonal effects of pregnancy and puberty hormones. However, there is little evidence in the literature to investigate this phenomenon. We propose that further studies need to be carried out to further examine this effect and to aid in managing keloids and hypertrophic scars during pregnancy. Women and girls who have keloid scarring should be made aware of this potential effect and should this occur need to seek the advice of a specialist to advise appropriately.
